# tRNA-Derived RNA Fragments Are Novel Biomarkers for Diagnosis, Prognosis, and Tumor Subtypes in Prostate Cancer

**DOI:** 10.3390/curroncol30010075

**Published:** 2023-01-10

**Authors:** Weigang Liu, Mengqian Yu, Sheng Cheng, Xiaoxu Zhou, Jia Li, Yan Lu, Pengyuan Liu, Shiping Ding

**Affiliations:** 1 Department of Cell Biology, The Children’s Hospital, Zhejiang University School of Medicine, Hangzhou 310058, China; 2 Key Laboratory of Precision Medicine in Diagnosis and Monitoring Research of Zhejiang Province, Sir Run Run Shaw Hospital and Institute of Translational Medicine, Zhejiang University School of Medicine, Hangzhou 310016, China; 3 Department of Urology, Sir Run Run Shaw Hospital and Institute of Translational Medicine, Zhejiang University School of Medicine, Hangzhou 310016, China; 4 Zhejiang Provincial Key Laboratory of Precision Diagnosis and Therapy for Major Gynecological Diseases, Department of Gynecologic Oncology, Women’s Hospital and Institute of Translational Medicine, Zhejiang University School of Medicine, Hangzhou 310006, China; 5 Cancer Center, Zhejiang University, Hangzhou 310013, China; 6 Department of Physiology and Center of Systems Molecular Medicine, Medical College of Wisconsin, Milwaukee, WI 53226, USA

**Keywords:** prostate adenocarcinoma, tRNA-derived RNA fragments, tumor subtypes, biomarker, diagnosis, prognosis

## Abstract

Background: tRNA-derived RNA fragments (tRFs) are a novel class of small ncRNA that are derived from precursor or mature tRNAs. Recently, the general relevance of their roles and clinical values in tumorigenesis, metastasis, and recurrence have been increasingly highlighted. However, there has been no specific systematic study to elucidate any potential clinical significance for these tRFs in prostate adenocarcinoma (PRAD), one of the most common and malignant cancers that threatens male health worldwide. Here, we investigate the clinical value of 5′-tRFs in PRAD. Methods: Small RNA sequencing data were analyzed to discover new 5′-tRFs biomarkers for PRAD. Machine learning algorithms were used to identify 5′-tRF classifiers to distinguish PRAD tumors from normal tissues. LASSO and Cox regression analyses were used to construct 5′-tRF prognostic predictive models. NMF and consensus clustering analyses were performed on 5′-tRF profiles to identify molecular subtypes of PRAD. Results: The overall levels of 5′-tRFs were significantly upregulated in the PRAD tumor samples compared to their adjacent normal samples. tRF classifiers composed of 13 5′-tRFs achieved AUC values as high as 0.963, showing high sensitivity and specificity in distinguishing PRAD tumors from normal samples. Multiple 5′-tRFs were identified as being associated with the PRAD prognosis. The tRF score, defined by a set of eight 5′-tRFs, was highly predictive of survival in PRAD patients. The combination of tRF and Gleason scores showed a significantly better performance than the Gleason score alone, suggesting that 5′-tRFs can offer PRAD patients additional and improved prognostic information. Four molecular subtypes of the PRAD tumor were identified based on their 5′-tRF expression profiles. Genetically, these 5′-tRFs PRAD tumor subtypes exhibited distinct genomic landscapes in tumor cells. Clinically, they showed marked differences in survival and clinicopathological features. Conclusions: 5′-tRFs are potential clinical biomarkers for the diagnosis, prognosis, and classification of tumor subtypes on a molecular level. These can help clinicians formulate personalized treatment plans for PRAD patients and may have similar potential applications for other disease types.

## 1. Introduction

Prostate adenocarcinoma (PRAD) is one of the most common and malignant cancers that threatens male health worldwide. In China, PRAD cases are increasing, with an estimated 125,646 new cases and 56,239 deaths in 2022 [1]. The lifetime probability of a PRAD diagnosis in men also increases significantly with age. Although the overall survival rate of PRAD is generally better than other cancers, its recurrence rate is also higher [2], and the majority of patients develop castration-resistant PRAD at advanced stages [3]. Despite great advances in the diagnosis and prognosis of PRAD [4], clinical parameters such as serum prostate-specific antigen (PSA) lack specificity for diagnosis and cannot definitively indicate individual prognosis [5]. Over recent years, increased numbers of other biomarkers have been used for the clinical diagnosis and prognosis of PRAD. Of note, urine levels of 8-OHdG and 8-Iso-PGF2α before and after surgery in patients with PRAD can help predict radicality (and possibly local recurrence) after robot-assisted radical prostatectomy [6]. However, clear biomarkers that facilitate accurate diagnosis, prognosis, and subtype classification are still lacking [7]. The identification of new, more definitive diagnostic and therapeutic biomarkers for PRAD would therefore be of great clinical significance.

tRNA-derived RNA fragments (tRFs) are a novel class of small noncoding RNAs (ncRNAs) that are derived from precursor or mature tRNAs. So far, many thousands of tRFs have been identified. These tRFs are broadly classified into six categories based on the cleavage sites at their parental tRNA: 5′-tRFs, 3′-tRFs, 5′-tRNA halves, 3′-tRNA halves, i-tRFs, and 3′U-tRFs (also known as tsRNAs or 1-tRF) [8,9]. RNase Z and ELAC2 cleave the 3′ end of the precursor tRNAs to generate 1-tRF [10]. The other three classes of tRFs are generated from different parts of mature tRNAs, with 5′ ends in the D-loop for 5′-tRFs, 3′ end in TψC loop for 3′-tRFs, and internal sites for i-tRFs [11]. In contrast, tRNA halves (including 5′-tRNA halves and 3′-tRNA halves) are generated from endonucleolytic cleavage of mature tRNA in the anti-code loop under angiogenin (ANG). These are also called tRNA-derived stress-induced RNAs [12].

Rather than random degradation products of tRNAs, tRFs are highly abundant and conserved across species, and their cleavages are site-specific [9]. Mounting evidence suggests that these small ncRNAs play important roles in cancer development and progression [13]. Aberrant expression of tRFs has been found to be involved in cell proliferation, invasive metastasis, and the progression of several human malignancies [9]. For example, CU1276 is involved in suppressing proliferation and modulating molecular responses to DNA damage by repressing endogenous RPA1 in B cell lymphoma [14]. tRF^Glu^, tRF^Asp^, tRF^Gly^, and tRF^Tyr^ can play tumor suppressive roles by destabilizing oncogenic transcripts by binding to YBX1 in breast cancer cells [15]. LeuCAG3′tsRNA can promote hepatocellular carcinogenesis by binding to two ribosomal protein mRNAs (RPS28 and RPS15) to enhance their translation [16]. Likewise, previous studies have also identified multiple tRFs involved in various aspects of prostate cancer [17]. tRNA-halves (also called SHOT-RNAs) from tRNA^Asp-GUC^, tRNA^His-GUG^, and tRNA^Lys-CUU^ have been noted as promoters of cellular proliferation in breast cancer and PRAD in a sex hormone-dependent manner [18]. tRF-1001 is required for cell proliferation, resulting from the cleavage of the Ser-TGA tRNA precursor transcript by tRNA 3′-endonuclease ELAC2 cutting in PRAD [8]. tRF-315, derived from tRNA-Lys-CTT, prevents cisplatin-induced apoptosis and attenuates cisplatin-induced mitochondrial dysfunction in PRAD cells [19]. The expression of tRFs is also affected during cancer development and progression, due to the activation of oncogenes and the inactivation of tumor suppressors [20,21].

In this regard, aberrantly expressed tRFs have great potential as new biomarkers for cancer diagnosis, prognosis, and tumor subtypes [9,13]. To assess the clinical significance of new tRF biomarkers for PRAD, this study analyzed small RNA sequencing data from 499 tumor tissues and 52 adjacent normal tissues of PRAD from The Cancer Genome Atlas (TCGA) dataset.

## 2. Methods

### 2.1. Data Collection

Small RNA sequencing datasets of 499 tumor tissues and 52 adjacent normal tissues, and PRAD somatic mutation datasets from TCGA, were downloaded from the Genomic Data Commons Portal (https://portal.gdc.cancer.gov/, accessed on 24 April 2021). The corresponding PRAD mRNA expression profiles (read counts) and patients’ clinical information, including survival time, age, and tumor stage, were downloaded from the International Cancer Genome Consortium (ICGC) Data Portal (https://dcc.icgc.org, accessed on 26 April 2021). Gene annotations and corresponding sequences for 610 nuclear tRNA genes in humans were downloaded from GtRNAdb (https://gtrnadb.ucsc.edu, accessed on 26 April 2021). The sequences and positions of 22 mitochondrial tRNA genes were downloaded from NCBI (https://www.ncbi.nlm.nih.gov/nuccore/251831106, accessed on 26 April 2021). All these datasets were further processed before subsequent analysis.

### 2.2. Identification and Quantification of 5′-tRFs in PRAD

A tRF annotation database for mapping and quantifying tRFs was built as previously described [22]. Using the created tRF annotation database, 5′-tRFs were detected and quantified from the small RNA sequencing datasets of PRAD. Briefly, reads in these BAM files were first remapped to sequence sets of our CCA-tRNA and pre-tRNA annotations using the burrows-wheeler transform (BWA) algorithm (http://biobwa.sourceforge.net/, accessed on 23 February 2021), allowing for no mismatches per read. These remapped reads were then used to count the number of reads belonging to each of the candidate 5′-tRFs. Finally, the expression level of 5′-tRFs was quantified as reads per million (RPM) of total mapped reads. To obtain robust 5′-tRFs, the 5′-tRFs with 90th quantile RPM < 1 were filtered, and those remaining were treated as detectable 5′-tRFs in PRAD. Furthermore, the expression level of 5′-tRFs was log2-transformed and then the upper-quantile normalized across samples before being used for downstream analysis.

### 2.3. Quantification of mRNA Expression Levels

mRNA expression levels from the corresponding PRAD samples in TCGA were normalized using the read per kilobase per million mapped reads (RPKM). Similarly, low-expressed genes with 90th quantile RPKM < 1 were removed and the remaining log_2_-transformed RPKM values were used for downstream analysis.

### 2.4. Identification of 5′-tRFs Dysregulated in PRAD

Expression levels of 5′-tRFs were compared between tumor tissues and adjacent normal tissues using a two-sample unpaired Wilcoxon rank sum test. Differentially expressed 5′-tRFs were detected using the Benjamini–Hochberg corrected *p*-value (i.e., false discovery rate, FDR) < 0.01 and |log_2_FC| > 1 between tumor and normal samples (Table S1). Next, the 13 most upregulated 5′-tRFs with |log_2_FC| > 2 and FDR < 0.01 were selected as tRF classifiers for the diagnosis of PRAD. Then, mathematical models were constructed to distinguish PRAD tumor tissues from normal tissues using different machine learning algorithms, such as random forest (RF), support vector machine (SVM), generalized linear model (GLM), and partial least squares (PLS). The receiver operation characteristic (ROC) was used to assess the sensitivity and specificity of the 5′-tRFs classifier at various classification thresholds. The area under the ROC curve (AUC) was calculated to evaluate the overall performance of tRF classifiers.

### 2.5. Inference of Potential Functions of 5′-tRFs

Studying the function of 5′-tRF is always problematic due to a lack of “prior” knowledge. To infer the functional role of dysregulated 5′-tRFs in PRAD, guilt by association (GBA) analysis was performed. Based on co-expression patterns, GBA has been widely used to study long non-coding RNAs [23]. Briefly, Pearson’s correlations (r) between expression of mRNA genes and dysregulated 5′-tRFs were estimated. mRNA genes co-expressed with dysregulated 5′-tRFs were identified when |r| > 0.3 and FDR < 0.05 were satisfied. Then, gene ontology (GO) analysis and Kyoto Encyclopedia of Genes and Genomes (KEGG) analyses of mRNA genes co-expressed with upregulated 5′-tRFs were performed using the web portal Metascape (https://metascape.org/ accessed on 28 March 2022).

### 2.6. Construction of Prognostic Predictor of 5′-tRFs

The association of 5′-tRF expression with PRAD prognosis was analyzed using the univariate Cox proportional hazards regression model. As a result, a set of 16 5′-tRFs that were significantly correlated with progression-free survival (PFS) was identified (*p*-value from both Wald test and log rank test <0.05). The hazard ratio (HR) and its 95% confidence interval (CI), *Z*-score, and *p*-value for each of these 16 5′-tRFs are listed in Table S2.

The least absolute shrinkage and selection operator (LASSO) was performed to select prognostic 5′-tRFs candidates using the R package ‘glmnet’ (v4.1-1). The optimal lambda was determined by 10-fold cross-validation, ultimately leading to the identification of 13 5′-tRFs by the LASSO analysis. These selected 5′-tRFs were further analyzed using a multivariate Cox regression model, of which eight 5′-tRFs remained statistically significant. To construct a prognostic model for PRAD, these eight 5′-tRFs were combined into one tRF score by summing their expression values multiplied by their corresponding coefficients in a multivariate Cox model. PRAD patients were divided into two groups according to the median tRF score. Kaplan–Meier curves of PFS and DFS were plotted for low and high tRF score groups, as implemented in the R package ‘survival’. The survival differences between two groups were assessed using a log rank test. The AUC metric was used to evaluate the overall performance of the tRF prognostic models for predicting 1-year, 3-year, and 5-year PFS and DFS using the R package ‘timeROC’ (v0.4). The association of clinical features (age, Gleason score, PSA, and the combination of Gleason and TS scores) with patient prognosis was also analyzed using the Cox regression models.

### 2.7. Identification of Tumor Subtypes Based on tRF Expression

The non-negative matrix factorization (NMF) method and consensus clustering analysis were performed on 5′-tRF expression profiles to identify molecular subtypes of PRAD. The consensus clustering with K-means method was then implemented in the R package ‘ConsensusClusterPlus’ (v1.58.0). Low-variation 5′-tRFs with interquartile ranges (IQRs) of <0.5 were filtered before NMF and cluster analyses. The optimal number of subtypes was determined according to the cophenetic and dispersion correlation coefficients. Kaplan–Meier curves of PFS and DFS were plotted for these 5′-tRFs expression subtypes. The log-rank test was used to evaluate the statistical differences in survival between different tRF subtypes.

### 2.8. Mutational Data Analysis

Segment files of PRAD derived from SNP 6.0 Affymetrix arrays were downloaded from TCGA. The segment files were divided into four categories (including tF-1, tF-2, tF-3, and tF-4), and then input into the online web portal Hiplot for the detection of amplification and deletion in each tumor’s genome (https://hiplot-academic.com/, accessed on 30 March 2022). The gene mutation data from PRAD were retrieved from the MAF file of TCGA, including single nucleotide variants (SNPs) and small insertions (INS) or deletions (DEL). These were then analyzed using the R package maftools (v. 2.10.5). The tumor mutational burden (TMB) was calculated by counting the number of non-synonymous somatic mutations per mega-base in protein-coding regions. DNA damages in PRAD tumor cells, including aneuploidy and homologous recombination deficiency (HRD), were quantitatively measured [24]. The HRD score was measured by summing three DNA-based measures of genomic instability: large (>15 Mb) non-arm-level regions with LOH, large-scale state transitions (breaks between adjacent segments of >10 Mb), and subtelomeric regions with allelic imbalance. The aneuploidy score (AS) for each tumor was measured by counting the number of arm-level gains and losses for a tumor, adjusted for ploidy [25].

### 2.9. Statistical Analysis

Continuous data were expressed as mean ± standard deviation (SD). Categorial data were presented as counts and frequencies. The Wilcoxon rank sum test was used to compare continuous variables between two groups. The Kruskal–Wallis H test was performed to compare continuous variables among more than two groups. The Chi-squared test was used to evaluate categorical data among several groups. *p* < 0.05 was considered statistically significant. For multiple comparisons, the Benjamini–Hochberg procedure was used for correcting *p*-values (i.e., FDR). All statistical analyses were performed using the R statistical package (v4.0.2).

## 3. Results

### 3.1. 5′- tRFs Are Dysregulated in PRAD

**Figure 1:**
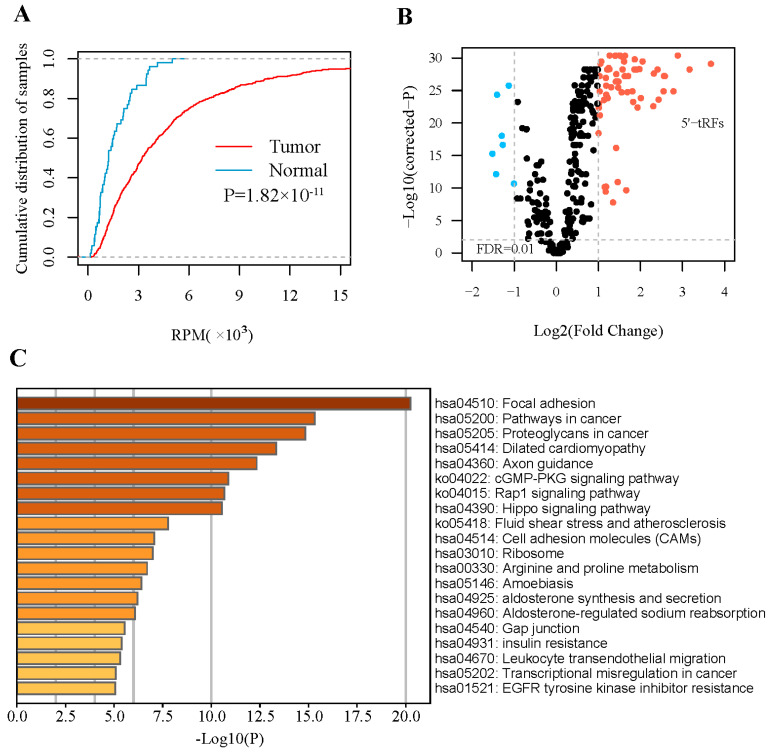
Expression of 5′-tRFs is dysregulated in PRAD. (**A**) Empirical cumulative distribution of the overall 5′-tRF in PRAD tumors and adjacent normal tissues. (**B**) Volcano plot showing −log10 (*p* value) vs. log2 (fold change) of 5′-tRFs between PRAD tumors and adjacent normal tissues. *p* values represent significance level of the difference in 5′-tRF expression between PRAD tumors and adjacent normal tissues. (**C**) Pathways enriched for mRNA genes co-expressed with significantly dysregulated 5′-tRFs.

We analyzed small RNA-sequencing data from 499 tumor tissues and 52 adjacent normal tissues of PRAD from TCGA. To obtain robust 5′-tRF profiles, 5′-tRFs with a 90th quantile RPM < 1 were filtered. As a result, 292 5′-tRFs were detected in these PRAD samples. Globally, 5′-tRFs tended to be upregulated in PRAD tumor samples compared to their adjacent normal samples (Figure 1A), of which 63 were significantly upregulated and 7 were significantly downregulated (FDR < 0.01 and |log2FC| > 1) (Figure 1B and Table S1). Whilst a similar analysis for 3′-tRFs was performed, very few 3′-tRFs were detected as differentially expressed between PRAD tumors and adjacent normal tissues and thus this research direction was not pursued further in our study.

To infer the functional roles of these upregulated 5′-tRFs, a causal association analysis based on co-expression patterns was performed for PRAD samples. It is well known that a group of co-expressed genes tend to have similar functions or be involved in common biological processes. This analysis yielded a total of 2008 protein-coding genes that were significantly co-expressed with these 5′-tRFs (FDR < 0.05 and |r| > 0.3). These genes were mainly enriched in areas of focal adhesion, cGMP-PKG, Rap1, and Hippo signaling (Figure 1C), and in several molecular functions such as glycosaminoglycan binding, kinase binding, and integrin binding (Figure S1). In other words, these upregulated 5′-tRFs were potentially involved in the above-mentioned signaling pathways and molecular functions.

### 3.2. 5′- tRFs Are Novel Biomarkers for Diagnosis of PRAD

**Figure 2:**
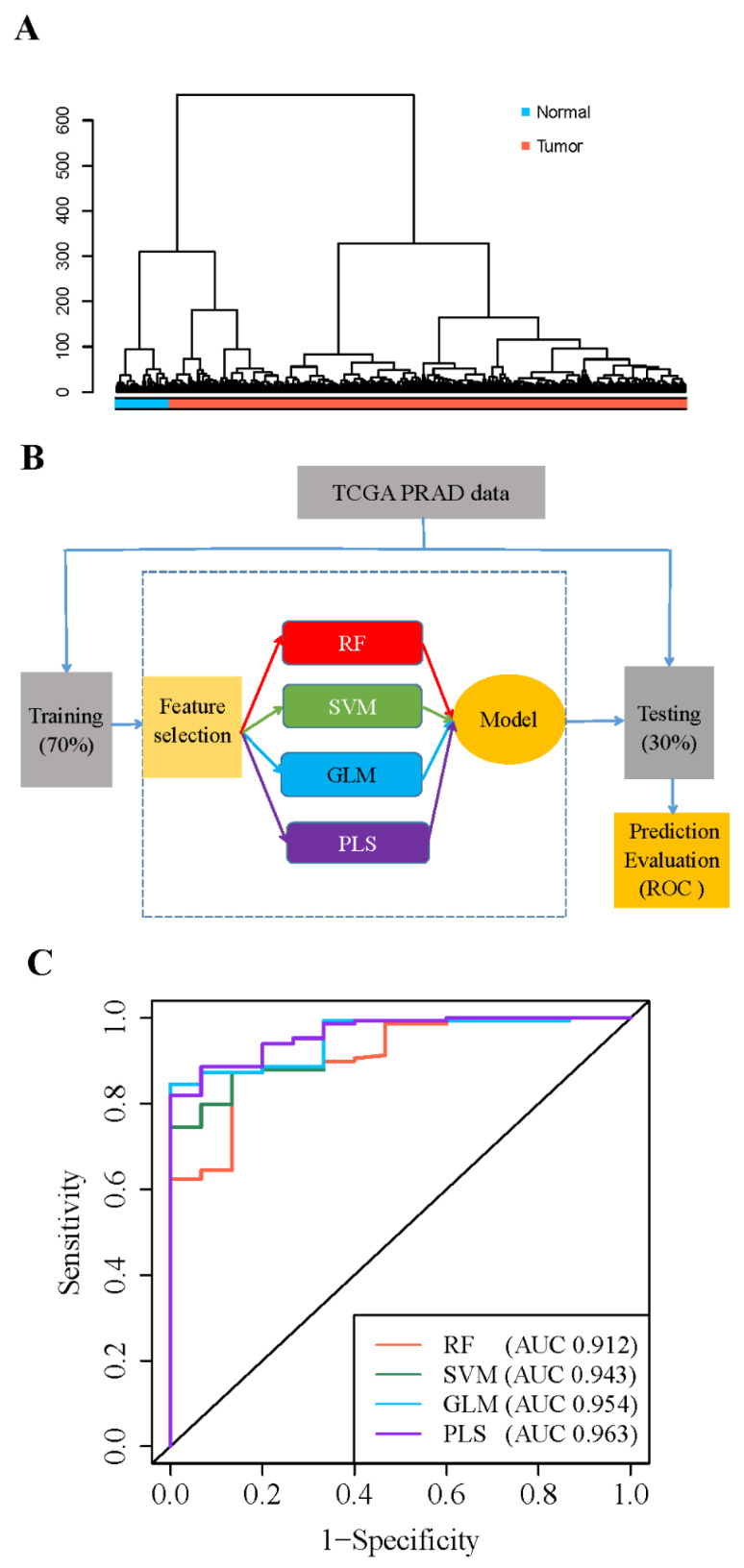
Expression of 5′-tRFs is a diagnostic biomarker for PRAD. (**A**) Hierarchical cluster analyses of PRAD specimens using 5′-tRFs expression profiles. (**B**) A schematic diagram for building machine learning models predictive of PRAD tumors versus normal tissues. RF: random forest; SVM: support vector machine; GLM: generalized linear model; PLS: partial least square. (**C**) ROC curves to evaluate the sensitivity and specificity of 5′-tRF classifiers to discriminate PRAD tumor and normal samples. AUC: the area under the ROC curve. When AUC = 1, the tRF classifier can perfectly distinguish between PRAD tumors and normal tissues.

5′-tRFs showed distinct expression patterns between PRAD tumors and adjacent normal tissues (Figure 2A and Figure S2). Of note, 63 of the 70 dysregulated 5′-tRFs were abnormally upregulated in PRAD tumors and thus considered to have great potential as clinical biomarkers for the diagnosis of PRAD. To evaluate whether these upregulated 5′-tRFs could indeed be used as clinical diagnostic biomarkers for PRAD, we selected the top 13 upregulated 5′-tRFs with fold changes >4 to build 5′-tRFs classifiers (Table S1). First, we randomly divided 551 PRAD tumor samples in TCGA into a training set (70%) and a testing set (30%). Next, we constructed mathematical models for the diagnosis of PRAD using four machine learning algorithms, including random forest (RF), support vector machine (SVM), generalized linear model (GLM), and partial least squares (PLS) analyses (Figure 2B). Then, the receiver operation characteristic (ROC) was used to assess the performance of the 5′-tRFs classifier at various classification thresholds. The area under the ROC curve (AUC) of the 5′-tRFs classifier defined by RF, GLM, SVM, or PLS achieved 0.912, 0.943, 0.954, or 0.963, respectively (Figure 2C). These results demonstrated that 5′-tRFs have great potential as valuable diagnostic biomarkers and that these 5′-tRFs classifiers consistently exhibit high sensitivity and specificity towards distinguishing PRAD tumor samples from normal samples.

### 3.3. 5′- tRFs Are Novel Biomarkers for Prognosis of PRAD

Next, we evaluated whether these 5′-tRFs could be used as clinical prognostic biomarkers for PRAD. Firstly, the association of each individual 5′-tRF with PRAD prognosis was examined using a univariate Cox regression model. Secondly, the identified 5′-tRFs with marginal significance (*p* < 0.05) were subjected to variable selection using the least absolute shrinkage and selection operator (LASSO) according to their prognostic predictive values (Figure S3A). This LASSO analysis identified a set of 5′-tRFs (13 5′-tRFs in total) that were strongly associated with clinical outcomes in PRAD (Table S2). Thirdly, this set of 5′-tRFs was further assessed using a multivariate Cox regression model. As a result, eight 5′-tRFs collectively showed significant prognostic values in predicting PFS in PRAD patients (Table S3 and Figure S3B).

**Figure 3:**
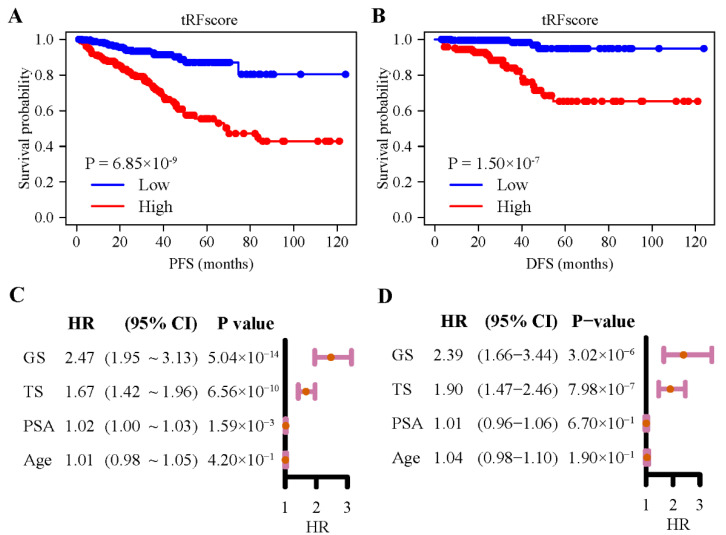
Expression of 5′-tRFs is a prognostic biomarker for PRAD. (**A**,**B**) Kaplan–Meier curves of PFS (**A**) and DFS (**B**) of PRAD patients with low vs. high tRF score. (**C**,**D**) Hazard ratios (HR) and their 95% confidence interval (CI) of GS, TS, PSA, and age from the univariate Cox regression model for PFS (**C**) and DFS (**D**). PFS: progression-free survival; DFS: disease-free survival; TS: tRF score; GS: Gleason score; PSA: serum prostate-specific antigen.

The eight 5′-tRFs were then combined into one tRF score (TS) as a prognostic signature for PRAD by summing their expression values multiplied by their corresponding coefficients in multivariate Cox models (Table S3). Kaplan–Meier survival curves were then plotted for groups of patients stratified by the median tRF score (Figure 3A,B). Patients with a lower tRF score had a significantly longer PFS (log rank test, *p* = 6.85 × 10^−9^) and DFS (*p* = 1.50 × 10^−7^) than those with a higher tRF score. For PFS, the hazard ratio for the tRF score reached 1.67 (CI, 1.42–1.96), while the hazard ratios for two common clinical parameters, the Gleason score (GS) and PSA, were 2.47 (1.95–3.13) and 1.02 (1.00–1.03), respectively (Figure 3C). For DFS, the hazard ratio for the tRF score reached 1.90 (1.47–2.46), while the hazard ratios for the two clinical parameters were 2.39 (1.66–3.44) and 1.01 (0.96–1.06), respectively (Figure 3D). When considering the associations of tRF score, Gleason score, or PSA with PRAD patient outcomes in the same multivariate Cox regression model, tRF score and Gleason scores remained statistically significant, but PSA did not (Figure S3C,D). These results suggested that 5′-tRFs are valuable prognostic biomarkers, and increased tRF scores are associated with poor prognosis in PRAD patients.

### 3.4. 5′- tRFs Provide Independent Prognostic Information for PRAD

**Figure 4:**
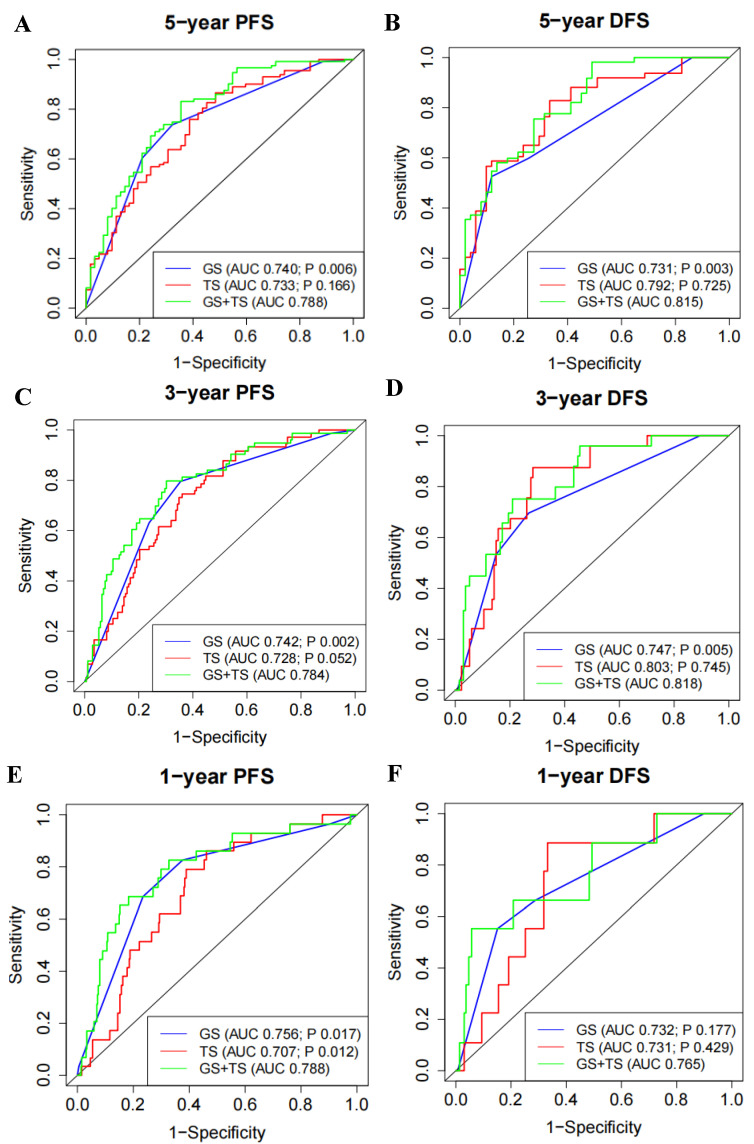
Expression of 5′-tRFs provides additional PRAD prognostic information independent of Gleason score. (**A**,**C**,**E**) ROC curves to evaluate the prognostic performance of 5-year (**A**), 3-year (**C**), and 1-year (**E**) PFS, using TS, GS, or TS and GS. (**B**,**D**,**F**) ROC curves to evaluate the prognostic performance of 5-year (**B**), 3-year (**D**), and 1-year (**F**) DFS, using TS, GS, or TS and GS. Bivariate prognostic models of GS and TS (i.e., GS and TS) were compared with univariate prognostic models to assess whether TS or GS could provide independent prognostic information. *p* value less than 0.05 indicates that the performance of the bivariate prognostic models of GS and TS (i.e., GS and TS) is significantly better than that of the univariate prognostic model. TS: tRF score; GS: Gleason score.

Furthermore, we evaluated whether 5′-tRF is suitable as an independent prognostic factor for PRAD. The AUC values of the tRF score, Gleason score, and PSA for predicting the risk of a 5-year PFS were 0.733, 0.740, and 0.571, respectively (Figure S4A). The AUC values of the three biomarkers for predicting the risk of a 5-year DFS were 0.792, 0.731, and 0.573, respectively (Figure S4B). A similar performance of these biomarkers was observed in predicting the risk of 1-year and 3-year PFS and DFS (Figure S4C–F). Interestingly, the combination of the tRF and Gleason scores showed significantly better performance than the Gleason score alone (Figure 4). For instance, the AUC values of the combined tRF and Gleason scores for predicting a 5-year PFS and DFS were 0.788 and 0.815, respectively, which were significantly higher than the corresponding AUC values of 0.740 (*p* = 0.006) and 0.731 (*p* = 0.003) for the Gleason score alone (Figure 4A,B). The superiority of the combination of tRF and Gleason scores was also demonstrated in predicting 1-year and 3-year PFS and DFS (Figure 4C–F).

For visualization purposes, nomogram models that incorporate the corresponding tRF and Gleason scores for predicting 1-year, 3-year, and 5-year PFS and DFS were established (Figure S4G,H). In these nomogram models, a probability of 1-year, 3-year, and 5-year PFS and DFS survival could be queried for PRAD patients. Taken together, these results suggested that 5′-tRFs are independent prognostic biomarkers and offer additional prognostic information independent of the Gleason score for PRAD patients.

### 3.5. 5′- tRFs Are Novel Biomarkers for the Tumor Classification of PRAD

**Figure 5:**
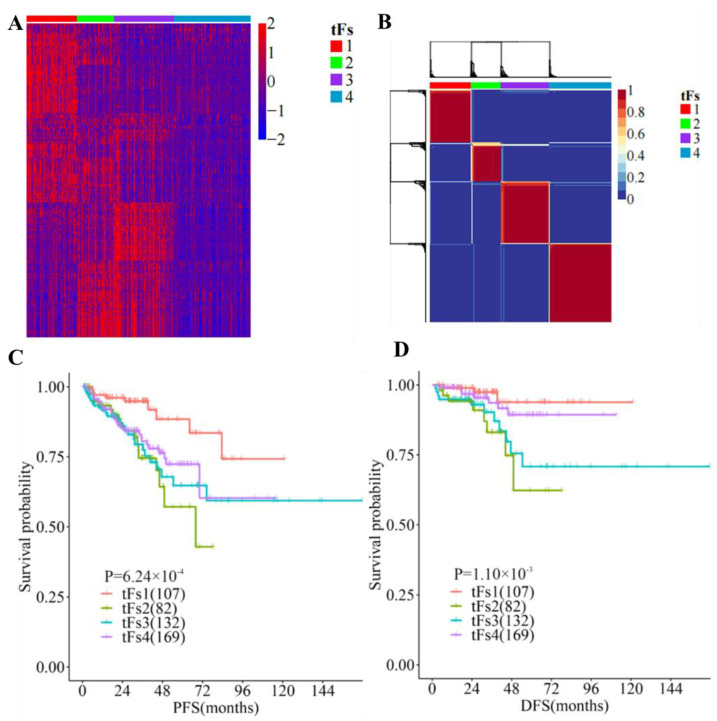
Expression of tRFs is a clinical biomarker for tumor classification in PRAD. (**A**) Analyses of 5′-tRFs in PRAD samples yielded four stable subgroups using a non-negative matrix factorization (NMF) approach. (**B**) Tumors were clustered into four subtypes according to 5′-tRF expression profiles. (**C**,**D**) Kaplan–Meier survival analysis of PFS (**C**) and DFS (**D**), showing significant prognostic differences among 5′-tRFs expression subtypes.

We then assessed whether these 5′-tRFs could be used as clinical biomarkers for the tumor classification of PRAD. We conducted a NMF clustering analysis for 5′-tRFs expression data to identify molecular subtypes and determined four clusters (termed as tF-1, *n* = 107; tF-2, *n* = 82; tF-3, *n* = 132; and tF-4, *n* = 169, respectively) as an optimal choice according to the cophenetic correlation coefficients, each of which has its own specific 5′-tRFs expression pattern (Figure 5A and Figure S5). Meanwhile, consensus clustering was also carried out on 5′-tRFs data. Interestingly, we found that the result of consensus clustering was consistent with that of the NMF method (Figure 5B). Furthermore, Kaplan–Meier survival analyses showed that there were significant differences in the survival times between these four PRAD molecular subtypes (*p* = 1.10 × 10^−3^ for DFS and *p* = 6.24 × 10^−4^ for PFS). Among these tRF subtypes, tF-1 had the best clinical outcome, while the tRF-2 had the worst clinical outcome, with tF-3 and tF-4 lying in a mid-range between the above two (Figure 5C,D).

In addition to their different clinical outcomes, these tRF subtypes also showed great variability in clinicopathological features such as PSA (Kruskal–Wallis H test, *p* = 0.02), Gleason score (Kruskal–Wallis H test, *p* = 2.07 × 10^−6^), and grade group (Chi-squared test, *p* = 3.93 × 10^−7^). Interestingly, the tF-1 subtype with the best prognosis tended to have a lower Gleason score, while the tF-2 with the worst prognosis tended to have a higher Gleason score. Similar trends were observed in grade groups for these tRF molecular subtypes. These findings indicated that 5′-tRFs are valuable biomarkers for the tumor classification of PRAD, and these molecular subtypes classified by 5′-tRFs exhibit significantly different clinicopathological characteristics (Table 1).

### 3.6. Genomic Landscapes of 5′-tRFs Tumor Subtypes of PRAD

**Figure 6:**
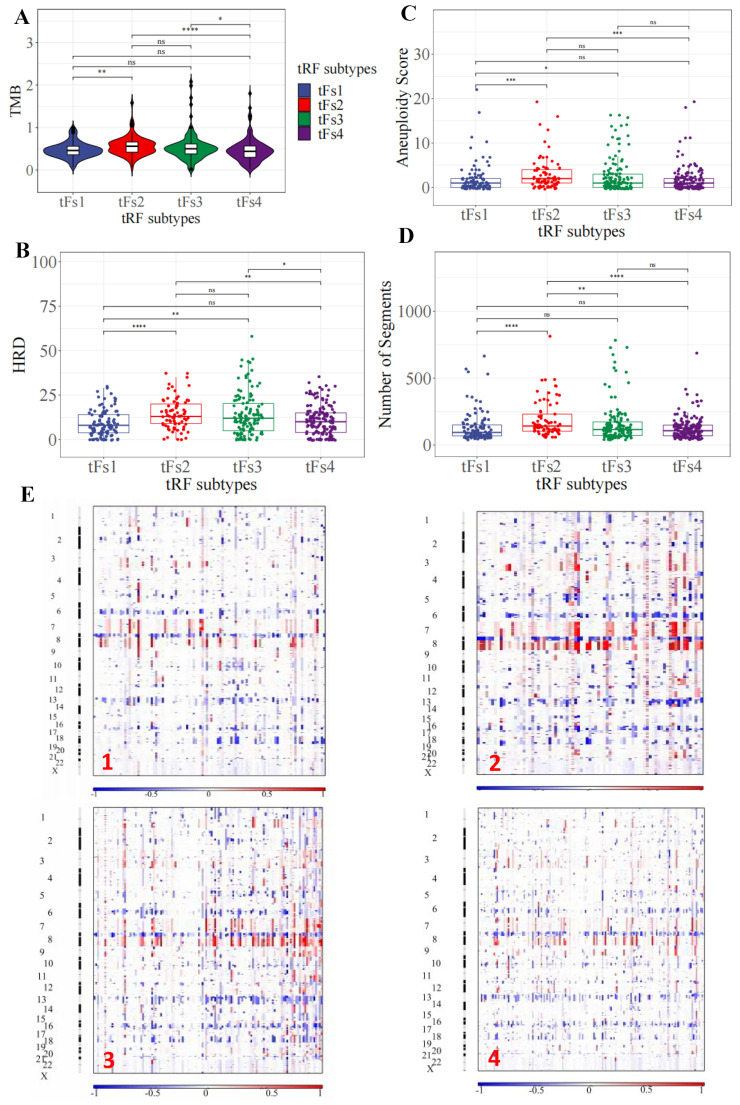
Distinct genomic landscapes among 5′-tRFs tumor subtypes. (**A**) Tumor mutation burden. The TMB was estimated by the number of non-synonymous somatic mutations (single nucleotide variants and small insertions/deletions) per Mb in protein-coding regions. (**B**) Homologous recombination defects. The HRD score was calculated by summing three DNA-based measures of genomic instability: large (>15 Mb) non-arm-level regions with LOH, large-scale state transitions (breaks between adjacent segments of >10 Mb), and subtelomeric regions with allelic imbalance. (**C**) Aneuploidy score. The AS is the total number of arm-level gains and losses for a tumor, adjusted for ploidy. (**D**) Number of segments. The number of segments was the total number of segments in each tumor’s copy number profile. * *p* < 0.05, ** *p* < 0.01, *** *p* < 0.001, **** *p* < 0.0001, ns: nonsignificant. (**E**) Somatic copy number variation. Chromosomes are represented along the vertical axis, samples are arranged horizontally; red indicates copy number amplification and blue indicates copy number loss.

To depict the genetic landscapes of these four 5′-tRFs PRAD subtypes, we analyzed somatic mutation data and somatic copy-number variation from TCGA. We first estimated the tumor mutational burden (TMB) by counting the number of non-synonymous somatic mutations per mega-base in the protein-coding regions. The tF-2 patients had the highest TMB, while the tF-1 and tF-4 patients had the lowest TMB among these 5′-tRF subtypes (Figure 6A). Next, we estimated homologous recombination defects (HRD), characterized by the inability of cells to effectively repair DNA double-strand breaks using the homologous recombination repair pathway [26]. Tumor cells from the tF-1 subtype were found to have fewer HRDs, while tumor cells from the tF-2 subtype had an increased number of HRDs (Figure 6B). We then estimated the aneuploidy score (AS) for each tumor by counting the number of arm-level gains and losses for a tumor, adjusted for ploidy. The tF-1 tumor subtype tended to have a low AS, while the tF-2 tumor subtype tended to have high a AS (Figure 6C). In addition, we estimated number of segmental duplications on each tumor genome. More segmental duplications were observed in tF-2 than in tF-1 (Figure 6D). Consistently, the tF-1 tumor subtype had much fewer copy-number variations than the tF-2 tumor subtype (Figure 6E and Figure S6). These results suggested that these 5′-tRFs tumor subtypes of PRAD exhibit distinct genomic landscapes in tumor cells. The best prognostic tF-1 subtype represented mild genomic alternations, and the worst prognostic tF-2 subtype represented a far more severe genomic alternation in tumor cells. The other two subtypes, tF-3 and tF-4, lay somewhere between the other two in this respect.

We also investigated whether these 5′-tRFs tumor subtypes of PRAD were driven by some key molecular events, such as androgen-regulated fusions of ERG or other ETS family members, or by other recurrent driver mutations. Interestingly, the tF-1 tumor subtype tended to have more frequent ETS family gene fusions than the tF-2 tumor subtype. These four 5′-tRFs tumor subtypes also showed significantly different frequencies of recurrent mutations in driver genes such as FOXA1 and KMT2D, with tF-2 having the highest frequency (Table 2). Taken together, these results suggested that these 5′-tRFs molecular subtypes are genomically distinct subgroups of PRAD. Understanding these genomically distinct alterations of these 5′-tRFs subtypes will therefore lead to a better diagnosis, prognosis, and treatment of PRAD.

## 4. Discussion

tRFs are a relatively newly discovered class of small ncRNAs that result from the precise cleavage of precursor or mature tRNAs by different types of nucleases. Over the recent years, their roles and clinical values in tumorigenesis, metastasis, and recurrence have attracted increasing attention [9]. However, there has been no systematic study to clarify the potential of these tRFs, especially 5′-tRFs, in the diagnosis, prognosis, and tumor classification of PRAD. In this study, we analyzed small RNA sequencing data to systematically assess the clinical values of 5′-tRFs in prostate adenocarcinoma. Our study demonstrated 5′-tRFs as promising clinical biomarkers for the diagnosis, prognosis, and classification of tumor molecular subtypes, which show strong potential to aid clinicians in developing personalized treatment plans for PRAD patients.

The overall levels of 5′-tRFs were significantly upregulated in the PRAD tumor samples compared to their adjacent normal samples. Interestingly, these aberrantly expressed 5′-tRFs were noted as those critically involved in many cancer-related pathways such as focal adhesion, cGMP-PKG, Rap1, and Hippo signaling, as well as in multiple related molecular functions such as glycosaminoglycan and integrin binding. For example, focal adhesion kinase is positively associated with the WHO grade group, tumor stage, Gleason score, perineural invasion, and extracapsular extension in PRAD [27]. Similarly, upregulation of the Hippo signaling effector YAP1 was noted to contribute to an earlier recurrence of PRAD [28]. It has also been reported that the increased expression of proteoglycans, including versican, biglycan, and syndecan-1, is associated with poor PRAD prognosis [29]. These abnormally upregulated 5′-tRFs in PRAD tumors therefore have great potential as clinical biomarkers for the diagnosis of PRAD. tRF classifiers composed of 13 such 5′-tRFs achieved AUC values as high as 0.963, showing high sensitivity and specificity in distinguishing PRAD tumor samples from normal samples.

In addition to serving as diagnostic biomarkers, multiple 5′-tRFs were identified as being associated with the PRAD prognosis. The tRF score, as defined by a set of 8 such 5′-tRFs, was highly predictive of PFS and DFS in PRAD patients. PRAD with high tRF scores tended to have a worse prognosis than those with low tRF scores. The Gleason scoring system, the most common prostate cancer grading system, is based on the extent to which the cancer looks like healthy tissue when viewed under a microscope. In addition to determining the tumor stage, the Gleason score helps the clinician tailor a patient-specific treatment plan. PRAD with low Gleason scores tends to be less aggressive and have better outcomes than those with high Gleason scores. The AUC values of the tRF score, Gleason score, and PSA for predicting the risk of 5-year PFS were 0.733, 0.740, and 0.571, respectively, while the AUC values of the three biomarkers for predicting the risk of 5-year DFS were 0.792, 0.731, and 0.573, respectively. Interestingly, the combination of the tRF and Gleason scores showed significantly better performance than the Gleason score alone, suggesting that 5′-tRFs can offer PRAD patients additional accuracy in prognostic information. Therefore, integrating the clinically commonly used Gleason score with the new biomarker tRF score will further improve individualized prognostic assessment and clinical decision-making in PRAD patients.

5′-tRFs can also serve as biomarkers for the molecular classification of tumor subtypes in PRAD. Four molecular subtypes of PRAD tumor were identified based on their 5′-tRF expression profiles. These tRF molecular subtypes exhibited marked differences in survival, with tRF-1 having the best outcome, tRF-2 having the worst, and both tRF-3 and tRF-4 having an intermediate prognosis. These subtypes classified by 5′-tRFs are also clinically relevant, characterized by different clinicopathological features. For example, the best prognostic tF-1 subtype tended to have a small Gleason score and a low-grade tumor, while the worst prognostic tF-2 tended to have a large Gleason score and a high-grade tumor.

These 5′-tRFs tumor subtypes of PRAD also bear distinct genomic landscapes in tumor cells. The tF-1 subtype with the best prognosis carried mild genomic alternations in tumor cells, while the tF-2 subtype with the worst prognosis carried much more severe genomic alternations in tumor cells. The other two subtypes, tF-3 and tF-4, lay intermediate between the two in this regard. Furthermore, these 5′-tRFs subtypes were driven by different key molecular events, such as androgen-regulated fusions of ERG and other ETS family members or recurrent driver mutations. For example, the tF-1 tumor subtype tended to have more frequent ETS family gene fusions than the tF-2 tumor subtype.

The molecular subtype classification of PRAD tumors by 5′-tRFs can not only provide clinicians with additional valuable prognostic information independent of the Gleason score but can also help clinicians formulate personalized treatment plans. Immunotherapy can improve the ability of the immune system to detect and destroy tumor cells. Over the recent years, many patients have benefited from immunotherapy, including some with metastatic cancers such as melanomas, lung cancer, and renal cell carcinoma. To compensate for the insufficiency of surgery and radiotherapy, immunotherapy was developed to try to alter the tumor immune-related microenvironment to treat PRAD [30]. However, only a minority of prostate cancer patients show positive responses to immunotherapy [30]. The tF-2 subtype has a higher TMB and more genomic alterations than the other subtypes and therefore may respond better to immunotherapy.

Furthermore, the tF-1 tumor subtype has more frequent ETS family gene fusions (such as ERG and ETV1) than the tF-2 tumor subtype. Therefore, the tRF-1 subtype may be more sensitive to, and therefore preferential for, androgen deprivation therapy than the tRF-2 subtype [31]. Recent preclinical studies have demonstrated an association between ETS gene fusions and components of the DNA damage response pathway [32]. Targeting DNA damage response pathways with inhibitors of PARP1, DNAPK, and HDAC1 may also be an alternative therapeutic option for the tRF-1 subtype with frequent EST gene fusions. HRD scores quantify the extent to which double-strand breaks in DNA in tumor cells cannot be repaired. Clinical trials have also shown that high levels of these HRD scores are associated with better responses to PARP inhibitor- or platinum-based therapy in ovarian and breast cancer [33,34]. Therefore, the tRF-2 subtype may respond better to PARP inhibitor- or platinum-based therapy than the other tRF subtypes in PRAD patients. A recent study showed that tRF-30-JZOYJE22RR33 and tRF-27-ZDXPHO53KSN are associated with trastuzumab resistance in breast cancer [35]. Whether these individual 5′-tRFs also play a role in resistance to PRAD treatments, such as androgen deprivation therapy, and whether they may be potential clinical biomarkers of drug sensitivity, remains to be further investigated.

Several caveats about our study should be acknowledged. Firstly, our tRF classifiers, survival prediction models, and tRF molecular subtypes were established based on the PRAD data from TCGA. These new findings require further validation from independent large clinical cohorts before they can be used clinically as diagnostic, prognostic, or subtyping biomarkers for PRAD. Although 5′-tRFs may provide additional and improved prognostic information for PRAD patients independent of the Gleason score, identifying which group of patients may benefit from the biopsy analysis of these biomarkers requires further study. In addition, developing clinically applicable PCR assays for these biomarkers is not trivial. Secondly, in this study we focused only on 5′-tRFs; other types of tRFs, including 3′-tRFs, should be further integrated into future investigations. Thirdly, tRFs are abundant and can be detected in bodily fluids such as blood samples, urine, saliva, and exosomes, making them promising non-invasive biomarkers for complex diseases such as cancer [9]. Therefore, the clinical value of these 5′-tRFs in the peripheral blood or urine of PRAD patients deserves further evaluation. Fourth, functional investigation of these dysregulated tRFs may help reveal novel mechanisms that underlie the development and progression of PRAD, and these warrant further research.

## 5. Conclusions

In summary, our study established a novel class of small ncRNAs-tRFs as potential clinical biomarkers for the diagnosis, prognosis, and classification of tumors in PRAD patients. These findings may not only provide clinicians with valuable diagnostic and prognostic information independent of the Gleason score but may also help clinicians formulate better treatment plans. Functional investigation of these dysregulated tRFs will help reveal novel mechanisms of PRAD development and progression.

## Figures and Tables

**Table 1:** Clinicopathological features of four tRF subtypes of PRAD.

	tF-1 (*n* = 107)	tF-2 (*n* = 82)	tF-3 (*n* = 132)	tF-4 (*n* = 169)	*p* Value
Age	60.60 ± 6.72	61.41 ± 6.37	61.60 ± 6.78	60.54 ± 7.01	0.744
PSA	9.22 ± 9.42	11.68 ± 8.83	12.74 ± 15.95	10.53 ± 11.8	0.020
Gleason score					2.07 × 10^−6^
6	17 (15.89%)	2 (2.44%)	13 (9.85%)	13 (7.69%)	
7	69 (64.49%)	36 (43.90%)	51 (38.64%)	87 (51.48%)	
8	13 (12.15%)	9 (10.98%)	17 (12.88%)	25 (14.79%)	
9/10	8 (7.47%)	35 (42.68%)	51 (38.63%)	44 (26.04%)	
Grading group					3.93 × 10^−7^
1	17 (15.89%)	2 (2.44%)	13 (9.85%)	13 (7.69%)	
2	47 (43.93%)	15 (18.29%)	31 (23.48%)	51 (30.18%)	
3	22 (20.56%)	21 (25.61%)	20 (15.15%)	36 (21.30%)	
4	13 (12.15%)	9 (10.98%)	17 (12.88%)	25 (14.79%)	
5	8 (7.47%)	35 (42.68%)	51 (38.64%)	44 (26.04%)	

**Table 2:** Key driver events in four tRF subtypes of PRAD.

	tF-1 (*n* = 89)	tF-2 (*n* = 52)	tF-3 (*n* = 38)	tF-4 (*n* = 147)	*p* Value
ERG (fusion)					1.24 × 10^−4^
Yes	34(38.20%)	12(23.08%)	16(42.10%)	84(57.14%)	
No	55(61.80%)	40(76.92%)	22(57.90%)	63(42.86%)	
FOXA1 (mutation)					
Yes	5(5.62%)	5(9.62%)	1(2.63%)	2(1.36%)	0.038
No	84(94.38%)	47(90.38%)	37(97.37%)	145(98.64%)	
KMT2D (mutation)					0.0018
Yes	1(1.12%)	7(13.46%)	1(2.63%)	2(1.36%)	
No	88(98.88%)	45(86.54%)	37(97.37%)	145(98.64%)	
ZMYM3 (mutation)					0.063
Yes	0(0.00%)	3(5.77%)	1(2.63%)	2(1.36%)	
No	89(100%)	49(94.23%)	37(97.37%)	145(98.64%)	

## Data Availability

The authors declare that all the data supporting the findings of this study are available within the Article.

## Supplementary Materials

The following supporting information can be downloaded at https://www.mdpi.com/article/10.3390/curroncol30010075/s1, Table S1: 5′-tRFs are differentially expressed between PRAD tumor and adjacent normal tissues. Table S2: univariate Cox regression analysis confirms 5′-tRF association with PRAD prognosis. Table S3: multivariate Cox regression analysis reveals eight 5′-tRFs showing significant association with PRAD prognosis. Figure S1: GO terms enriched for mRNA genes that were co-expressed with significantly dysregulated 5′-tRFs. Figure S2: hierarchical cluster analyses of PRAD specimens using 5′-tRF expression profiles. Figure S3: 5′-tRFs are associated with the prognosis of PRAD. Figure S4: prognostic performance of the Gleason score, tRF score, PSA, and age. Figure S5: determining an optimal number of tRF subtypes in PRAD. Figure S6: copy number gain and loss in 5′-tRFs tumor subtypes.

## Abbreviations

tRFs	tRNA-derived RNA fragments
PRAD	prostate adenocarcinoma
PSA	serum prostate-specific antigen
ncRNAs	small noncoding RNAs
ANG	angiogenin
TCGA	The Cancer Genome Atlas
ICGC	International Cancer Genome Consortium
BWA	burrows-wheeler transform
RPM	reads per million
RPKM	read per kilobase per million
FDR	false discovery rate
RF	random forest
SVM	support vector machine
GLM	generalized linear model
PLS	partial least squares
ROC	receiver operation characteristic
GBA	guilt by association
GO	gene ontology
KEGG	Kyoto encyclopedia of genes and genomes
PFS	progression-free survival
HR	hazard ratio
CI	confidence interval
LASSOl	east absolute shrinkage and selection operator
NMF	non-negative matrix factorization
IQRs	interquartile ranges
SNP	single nucleotide polymorphism
INS	small insertions
DEL	deletions
TMB	tumor mutational burden
HRD	homologous recombination deficiency
AS	aneuploidy score
SD	standard deviation
TS	tRF score
GS	Gleason score
DFS	disease-free survival
