# Supplementary material for: tRNA-Derived RNA Fragments Are Novel Biomarkers for Diagnosis, Prognosis, and Tumor Subtypes in Prostate Cancer
Source: Curr Oncol. 2023 Jan 10;30(1):981–99. doi: 10.3390/curroncol30010075 (PMC9857875; doi:10.3390/curroncol30010075)
Supplement: Supplementary file 1 [file curroncol-30-00075-s001.zip › curroncol-2111223-supplementary.pdf]

tRNA-derived RNA fragments are novel biomarkers for diagnosis, prognosis, and tumor subtypes in prostate cancer

Supplementary Materials

Table S1 5′-tRFs are differentially expressed between PRAD tumor and adjacent normal tissues*

tRFs	Log(FC)	P value
Upregulated		
**5'-M-tRNA-Glu-TTC-1-2_L30**	3.676	8.74E-31
**5'-M-tRNA-Glu-TTC-2-2_L30**	3.166	2.18E-29
**5'-M-tRNA-Gly-GCC-5-1_L30**	2.888	6.07E-90
**5'-M-tRNA-Gly-GCC-5-1_L29**	2.787	3.95E-26
**5'-M-tRNA-Val-CAC-5-1_L30**	2.581	1.37E-28
**5'-M-tRNA-Gly-GCC-5-1_L28**	2.56	1.86E-28
**5'-M-tRNA-Glu-CTC-2-1_L29**	2.551	1.99E-26
**5'-M-tRNA-Gly-GCC-1-5_L30**	2.432	3.30E-25
**5'-M-tRNA-Glu-CTC-2-1_L30**	2.405	1.07E-29
**5'-M-tRNA-His-GTG-1-9_L30**	2.32	4.50E-29
**5'-M-tRNA-Gln-CTG-5-1_L30**	2.306	3.26E-24
**5'-M-tRNA-Leu-CAG-2-2_L30**	2.051	3.64E-31
**5'-M-tRNA-Cys-GCA-4-1_L29**	2.01	2.74E-25
5'-M-tRNA-Gln-TTG-4-1_L30	1.978	6.40E-30
5'-M-tRNA-Tyr-GTA-chr21-2_L30	1.932	8.47E-24
5'-M-tRNA-Gln-CTG-5-1_L29	1.918	2.24E-29
5'-M-tRNA-Ala-TGC-4-1_L24	1.865	1.23E-31
5'-tRNA-Gly-TCC-1-1_L30	1.857	2.82E-29
5'-M-tRNA-Gly-CCC-2-2_L29	1.828	1.37E-24
5'-M-tRNA-Gly-GCC-5-1_L27	1.809	3.03E-26
5'-M-tRNA-Gln-TTG-3-3_L30	1.709	1.10E-28
5'-M-tRNA-Lys-TTT-5-1_L30	1.69	5.22E-26
5'-M-tRNA-Arg-TCG-1-1_L19	1.663	1.42E-11
5'-M-tRNA-Leu-TAG-1-1_L26	1.631	6.42E-32
5'-M-tRNA-Ser-TGA-4-1_L24	1.627	1.33E-28
5'-M-tRNA-Tyr-GTA-3-1_L25	1.612	4.58E-31
5'-M-tRNA-Pro-TGG-3-5_L30	1.576	3.61E-29
5'-M-tRNA-Ser-TGA-4-1_L25	1.558	9.97E-32
5'-M-tRNA-Glu-CTC-2-1_L28	1.555	5.34E-26
5'-M-tRNA-Leu-CAG-2-2_L25	1.511	3.38E-32
5'-M-tRNA-Val-TAC-1-2_L30	1.478	8.31E-28
5'-M-tRNA-Leu-TAG-1-1_L30	1.473	6.35E-27
5'-M-tRNA-Val-CAC-5-1_L23	1.469	8.03E-28
5'-M-tRNA-Leu-TAG-1-1_L21	1.462	1.45E-12
5'-tRNA-Val-CAC-8-1_L30	1.426	5.45E-29
5'-M-tRNA-Gly-GCC-1-5_L29	1.422	4.97E-18
5'-M-tRNA-Tyr-GTA-7-1_L25	1.416	4.03E-28
5'-M-tRNA-Leu-TAG-1-1_L25	1.412	3.20E-32
5'-M-tRNA-Ser-GCT-5-1_L24	1.398	9.71E-32
5'-tRNA-Arg-TCT-1-1_L19	1.349	1.08E-09
5'-M-tRNA-Gly-TCC-4-1_L23	1.305	5.11E-27

5'-tRNA-Leu-TAA-1-1_L25	1.269	4.82E-32
5'-M-tRNA-iMet-CAT-2-1_L30	1.264	2.54E-30
5'-M-tRNA-Leu-CAG-2-2_L26	1.246	1.18E-30
5'-tRNA-Pro-TGG-2-1_L30	1.234	3.29E-29
5'-M-tRNA-Glu-TTC-2-2_L28	1.221	2.54E-25
5'-tRNA-Glu-TTC-9-1_L30	1.209	1.54E-29
5'-M-tRNA-Lys-CTT-3-1_L30	1.194	1.59E-25
5'-M-tRNA-Thr-CGT-4-1_L24	1.187	3.03E-27
5'-tRNA-Arg-CCG-2-1_L30	1.185	8.86E-26
5'-M-tRNA-Arg-ACG-2-4_L19	1.184	6.47E-12
5'-M-tRNA-Lys-CTT-4-1_L30	1.181	3.57E-28
5'-tRNA-Val-AAC-2-1_L20	1.174	3.19E-11
5'-M-tRNA-Arg-TCT-5-1_L19	1.148	1.14E-11
5'-M-tRNA-Gly-TCC-2-6_L30	1.136	4.30E-25
5'-M-tRNA-Leu-TAG-1-1_L24	1.069	1.86E-31
5'-M-tRNA-Ser-CGA-2-1_L25	1.052	5.89E-31
5'-tRNA-Val-TAC-2-1_L30	1.036	4.94E-23
5'-tRNA-Val-AAC-2-1_L23	1.031	7.78E-27
5'-M-tRNA-Gln-CTG-5-1_L23	1.029	6.64E-29
5'-M-tRNA-Leu-TAG-1-1_L22	1.018	2.59E-24
5'-M-tRNA-Ala-TGC-4-1_L22	1.013	1.17E-23
5'-M-tRNA-Tyr-GTA-7-1_L23	1.006	4.27E-20
Dowregulated		
5'-M-tRNA-Leu-TAG-3-1_L15	-1.005	4.38E-12
5'-M-tRNA-Glu-TTC-1-2_L16	-1.129	4.85E-27
5'-M-tRNA-Val-TAC-3-1_L15	-1.271	2.59E-18
5'-M-tRNA-Ser-TGA-4-1_L18	-1.296	2.58E-19
5'-M-tRNA-Gly-CCC-2-2_L15	-1.409	6.11E-26
5'-M-tRNA-Ser-TGA-4-1_L15	-1.431	1.63E-13
5'-M-tRNA-Val-CAC-7-1_L15	-1.518	1.38E-17

*Note: The top 13 upregulated 5'-tRFs (highlighted by bold font) were used to build tRF classifiers for diagnosis of PRAD.

Table S2 Univariate Cox regression analysis confirms 5'-tRF association with PRAD prognosis*

tRF	Z-score	HR	Low95%CI	High95%CI	Wald test	Log-rank test
**5'-tRNA-Arg-TCG-3-1_L18**	-2.79	0.49	0.30	0.81	0.005	0.001
**5'-M-tRNA-Val-AAC-6-1_L20**	-2.21	0.60	0.39	0.95	0.027	0.003
**5'-M-tRNA-Glu-TTC-1-2_L16**	-2.56	0.48	0.28	0.84	0.01	0.004
**5'-tRNA-Glu-TTC-9-1_L30**	2.16	1.47	1.04	2.07	0.031	0.006
**5'-M-tRNA-Ser-TGA-4-1_L15**	-2.36	0.75	0.59	0.95	0.018	0.007
**5'-M-tRNA-Leu-TAG-1-1_L17**	-2.84	0.36	0.18	0.73	0.005	0.010
**5'-M-tRNA-Arg-CCT-4-1_L18**	-2.74	0.60	0.42	0.87	0.006	0.014
**5'-M-tRNA-Ala-TGC-4-1_L23**	2.15	1.29	1.02	1.63	0.032	0.016
**5'-M-tRNA-Lys-TTT-5-1_L22**	2.49	1.73	1.12	2.65	0.013	0.024
5'-tRNA-Arg-TCG-3-1_L19	-1.98	0.83	0.68	1.00	0.048	0.025
**5'-M-tRNA-Ala-TGC-4-1_L16**	-2.60	0.49	0.29	0.84	0.009	0.028
5'-M-tRNA-Arg-TCG-1-1_L18	-2.24	0.74	0.57	0.96	0.025	0.032
**5'-M-tRNA-Ala-TGC-4-1_L24**	2.09	1.34	1.02	1.77	0.037	0.034
**5'-M-tRNA-Gly-GCC-1-5_L22**	2.00	1.43	1.01	2.04	0.046	0.037
5'-M-tRNA-Gly-TCC-4-1_L18	-2.41	0.63	0.43	0.92	0.016	0.042
**5'-M-tRNA-Lys-TTT-5-1_L23**	2.64	1.79	1.16	2.76	0.008	0.05

*Note: These identified 5'-tRFs with marginal significance were further subjected to variable selection using LASSO (the one highlighted by bold font).

Table S3 Multivariate Cox regression analysis reveals eight 5'-tRFs showing significant association with PRAD prognosis*

tRFs	coef	HR	Z-score	P value	Low95%CI	High95%CI
5'-tRNA-Arg-TCG-3-1_L18	-0.4808	0.6183	-1.4631	0.1434	0.3247	1.1774
5'-M-tRNA-Glu-TTC-1-2_L16	-0.8764	0.4163	-3.0229	0.0025	0.2358	0.7348
5'-tRNA-Glu-TTC-9-1_L30	0.3224	1.3805	1.6809	0.0928	0.9479	2.0106
5'-M-tRNA-Leu-TAG-1-1_L17	-0.5624	0.5698	-1.5320	0.1255	0.2775	1.1701
5'-M-tRNA-Arg-CCT-4-1_L18	-0.3822	0.6824	-1.5064	0.1320	0.4150	1.1220
5'-M-tRNA-Ala-TGC-4-1_L23	0.2152	1.2402	1.6799	0.0930	0.9648	1.5942
5'-M-tRNA-Lys-TTT-5-1_L22	0.7238	2.0623	2.8348	0.0046	1.2503	3.4018
5'-M-tRNA-Ala-TGC-4-1_L16	-0.4262	0.6530	-1.4640	0.1432	0.3691	1.1553

*Note: Coefficients estimated from the multivariate Cox regression model were used to calculate tRF score.

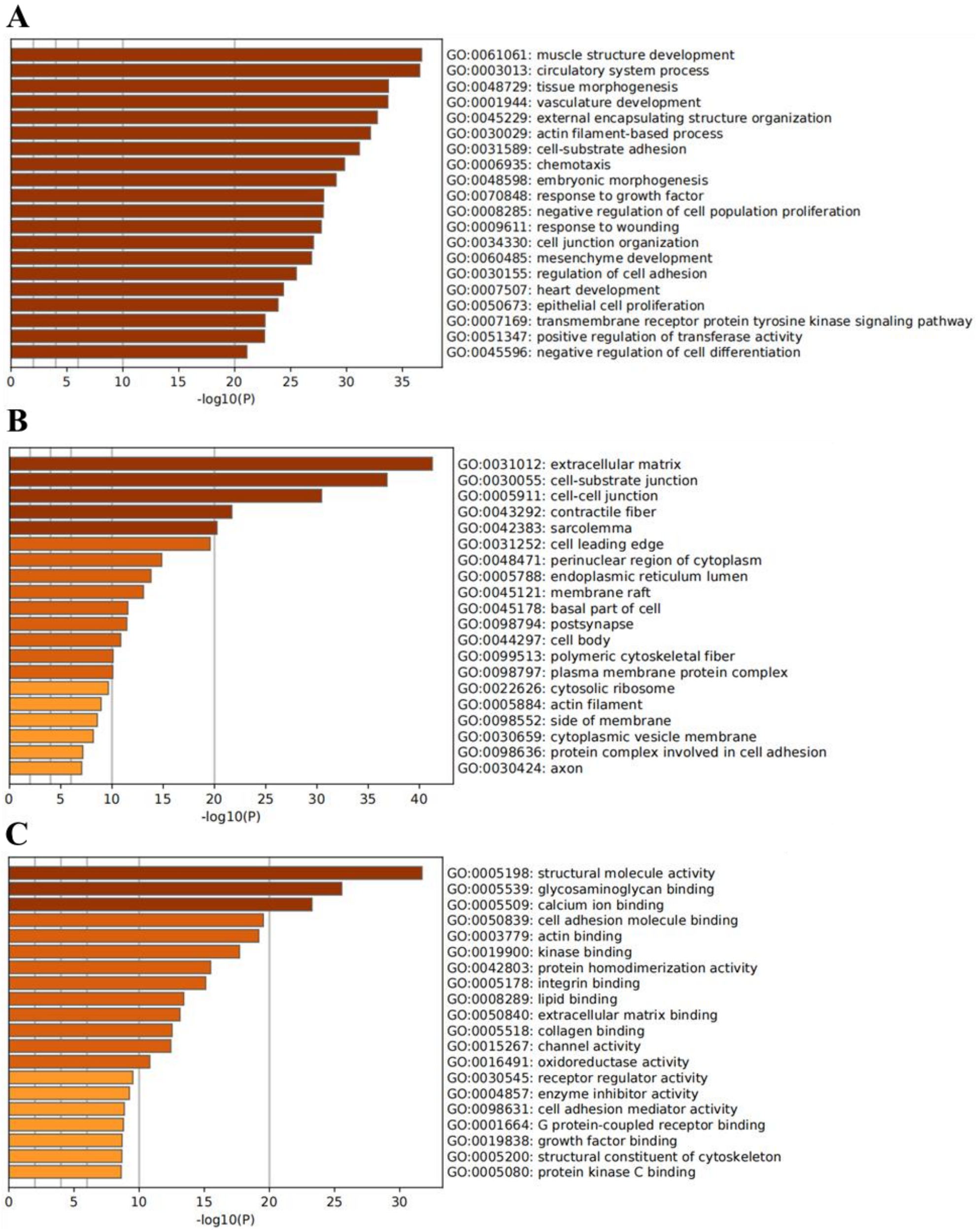

Figure S1 GO terms enriched for mRNA genes that were co-expressed with significantly dysregulated 5′-tRFs. Related to Figure 1. (**A**) Biological process. (**B**) Cellular component. (**C**) Molecular function.

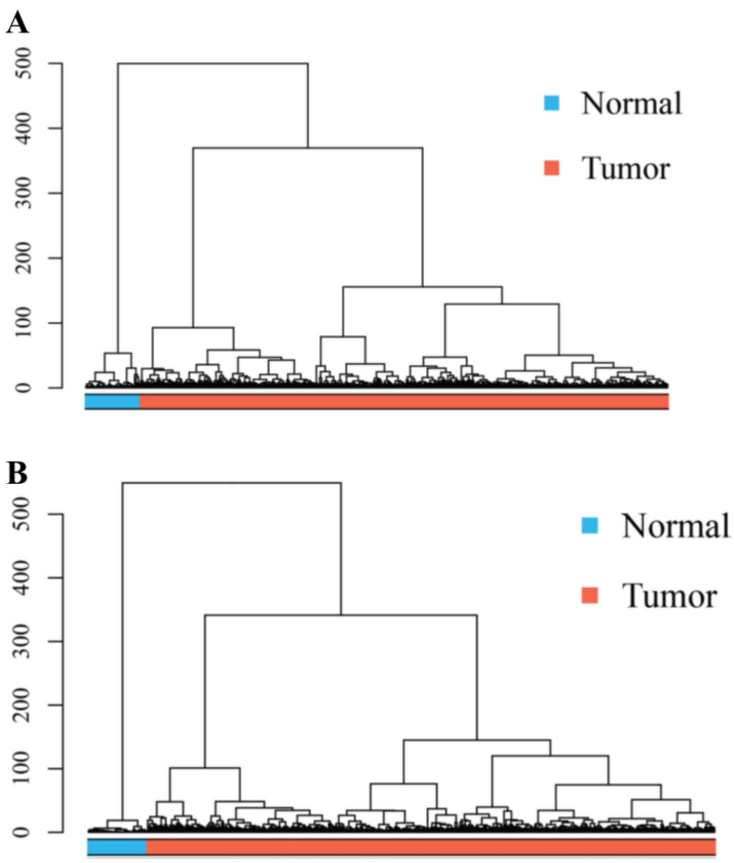

Figure S2 Hierarchical cluster analyses of PRAD specimens using 5′-tRF expression profiles. Related to Figure 2. (**A**) 70 5′-tRFs differentially expressed between PRAD tumor and adjacent normal tissues. (**B**) 63 5′-tRFs upregulated in PRAD tumors.

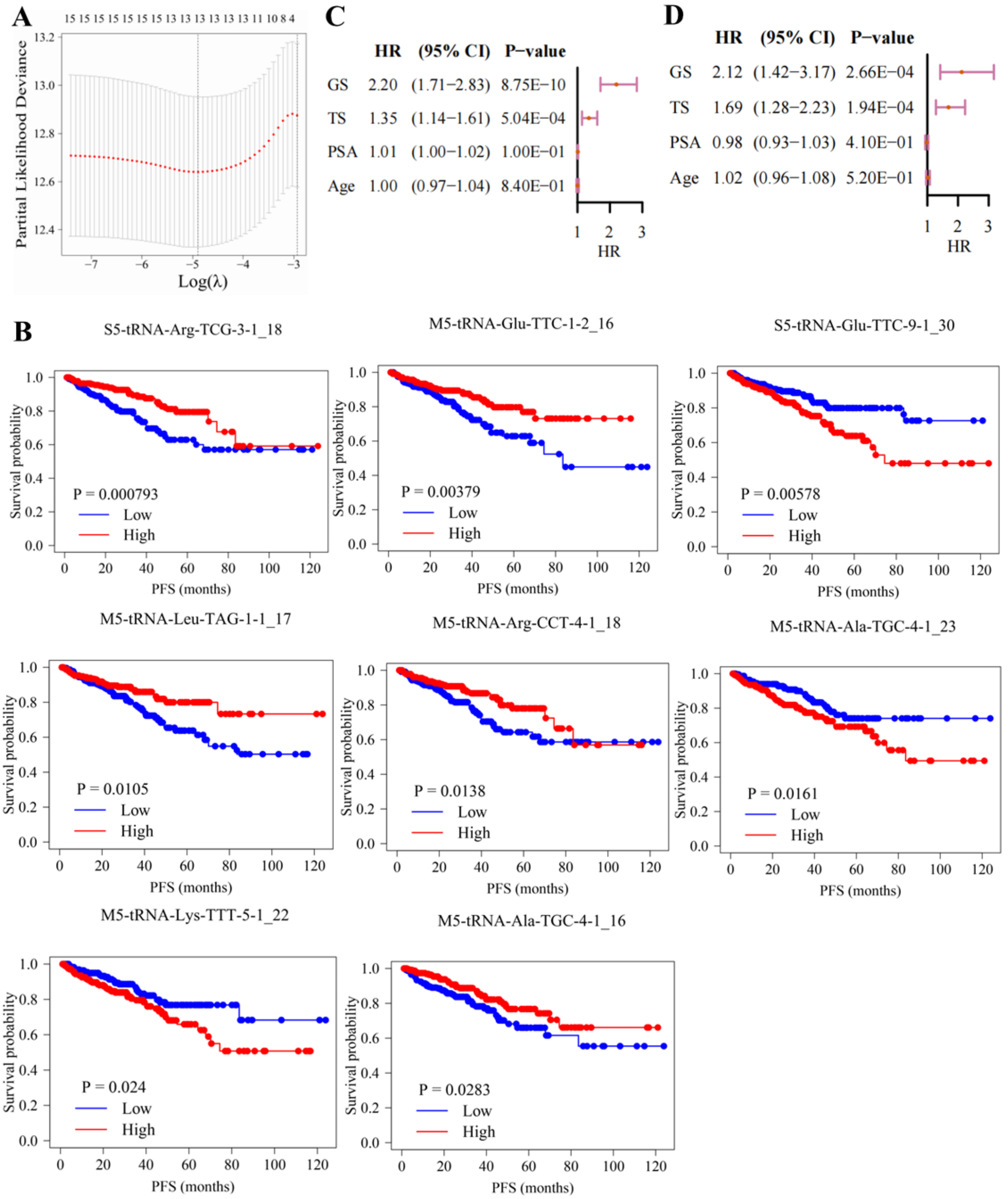

Figure S3 5′-tRFs are associated with the prognosis of PRAD. Related to Figure 3. (**A**) Partial likelihood deviance versus lambda relationship in LASSO. According to the partial likelihood deviance, a subset of 13 5′-tRF that minimizes prediction error of PFS were identified. (**B**) Kaplan-Meier curves of PFS of PRAD patients with low vs high 5′-tRF expression. Eight 5′-tRFs included in the tRF score were analyzed separately, including S5-tRNA-Arg-TCG-3-1_18, M5-tRNA-Glu-TTC-1-2_16, M5-tRNA-Leu-TAG-1-1_17, M5-tRNA-Arg-CCT-4-1_18, M5-tRNA-Ala-TGC-4-1_16, S5-tRNA-Glu-TTC-9-1_30, M5-tRNA-Ala-TGC-4-1_23, and M5-tRNA-Lys-TTT-5-1_22. (**C, D**) HRs and their 95% CI of GS, TS, PSA and age estimations from the multivariate Cox regression model for PFS (**C**) and DFS (**D**).

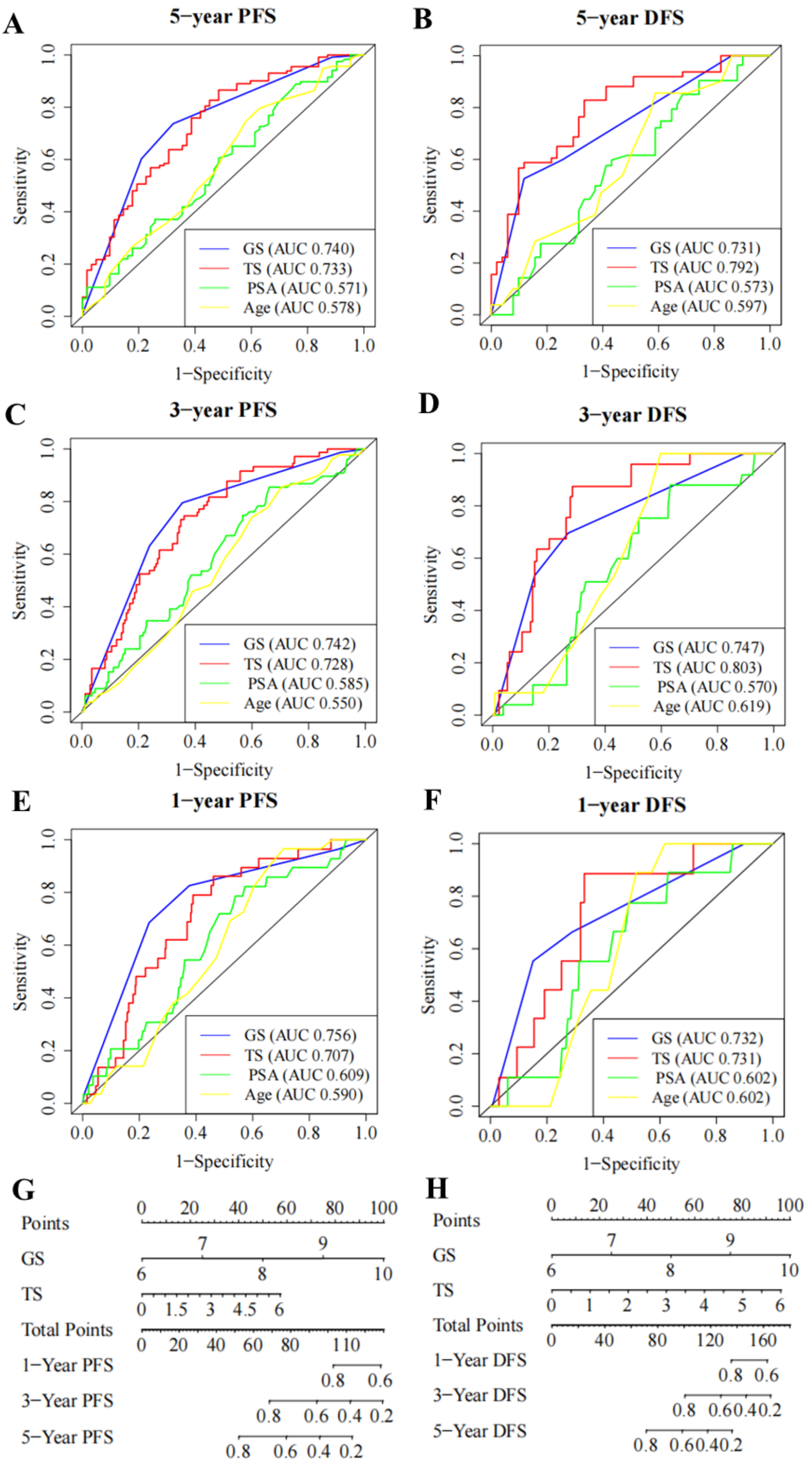

Figure S4 Prognostic performance of Gleason score, tRF score, PSA and age. Related to Figure 4. (**A, C, E**) ROC curves to evaluate the prognostic performance of 5-year (**A**), 3-year (**C**) and 1-year (**E**) PFS using TS, GS, PSA, or age. (**B, D, F**) ROC curves to evaluate the prognostic performance of 5-year (**B**), 3-year (**D**) and 1-year (**F**) DFS using TS, GS, PSA, or age. (**G, H**) Nomograms to estimate the risk of PFS (**G**) and DFS (**H**) in PRAD patients.

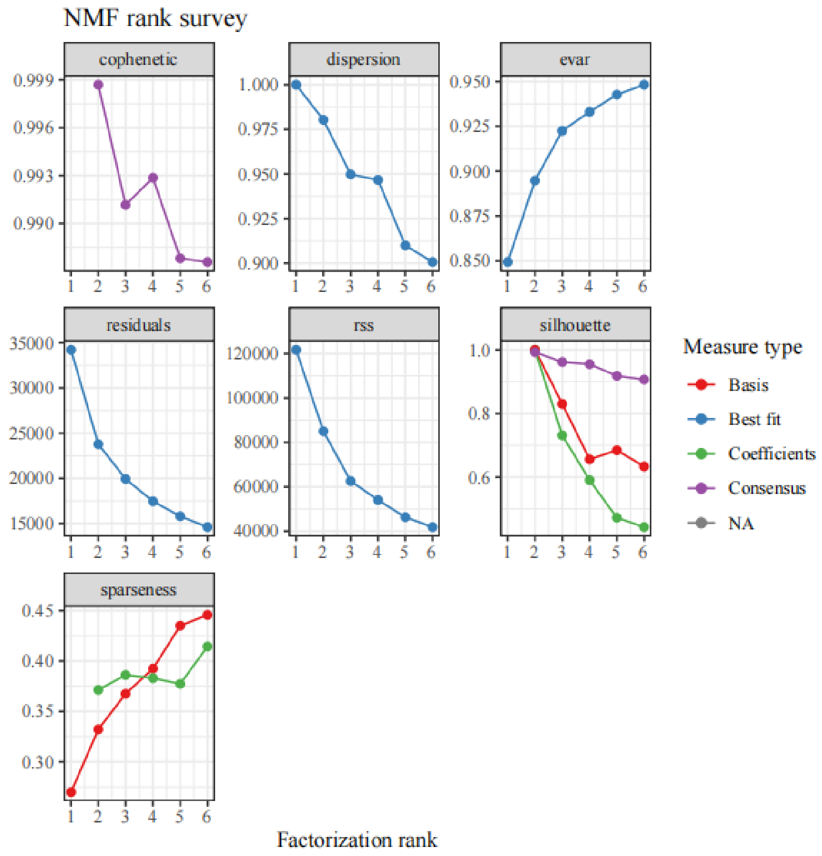

Figure S5 Determining an optimal number of tRF subtypes PRAD in HNSC. Related to Figure 5. The cophenetic correlation coefficient is a measure of the dispersion of the consensus matrix based on the average of connectivity matrices. It measures the stability of the 5′-tRF clusters obtained from NMF analysis of 5′-tRF expression data in PRAD.

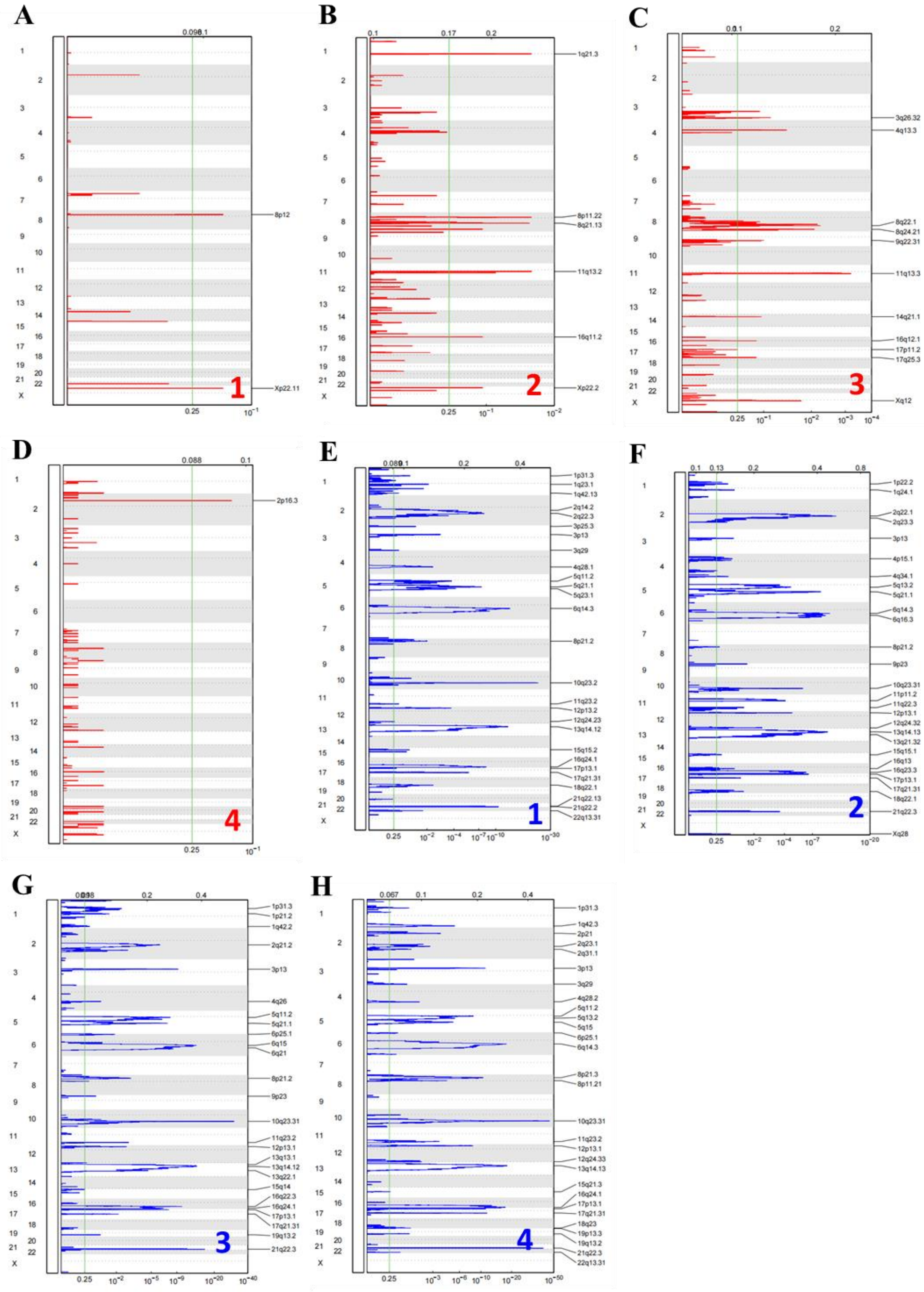

Figure S6 Copy number gain and loss in 5′-tRFs tumor subtypes. Related to Figure 6. (**A-D**) Somatic gain of copy number in four 5′-tRFs tumor subtypes. (**E-H**) Somatic loss of copy number in four 5′-tRFs tumor subtypes. The numbers 1, 2, 3 and 4 at the lower right corner of each panel indicate 5′-tRFs tumor subtypes, tRF-1, tRF-2, tRF-3, and tRF-4, respectively.
